# The intervening domain from MeCP2 enhances the DNA affinity of the methyl binding domain and provides an independent DNA interaction site

**DOI:** 10.1038/srep41635

**Published:** 2017-01-31

**Authors:** Rafael Claveria-Gimeno, Pilar M. Lanuza, Ignacio Morales-Chueca, Olga C. Jorge-Torres, Sonia Vega, Olga Abian, Manel Esteller, Adrian Velazquez-Campoy

**Affiliations:** 1 Institute of Biocomputation and Physics of Complex Systems (BIFI), Joint Units IQFR-CSIC-BIFI, and GBsC-CSIC-BIFI, Universidad de Zaragoza, Zaragoza, 50018, Spain; 2 Instituto Aragonés de Ciencias de la Salud (IACS), Zaragoza, 50009, Spain; 3 Aragon Institute for Health Research (IIS Aragon), Zaragoza, 50009, Spain; 4 Department of Biochemistry and Molecular and Cell Biology, Universidad de Zaragoza, Zaragoza, 50009, Spain; 5 Cancer Epigenetics and Biology Program (PEBC), Bellvitge Biomedical Research Institute (IDIBELL), Hospitalet de Llobregat, Barcelona, 08908, Spain; 6 Centro de Investigación Biomédica en Red en el Área Temática de Enfermedades Hepáticas y Digestivas (CIBERehd), Barcelona, Spain; 7 Department of Physiological Sciences II, School of Medicine, University of Barcelona, L’Hospitalet de Llobregat, Barcelona, 08907, Spain; 8 Institucio Catalana de Recerca i Estudis Avançats, Barcelona, 08010, Spain; 9 Fundacion ARAID, Government of Aragon, Zaragoza, 50018, Spain

## Abstract

Methyl-CpG binding protein 2 (MeCP2) preferentially interacts with methylated DNA and it is involved in epigenetic regulation and chromatin remodelling. Mutations in MeCP2 are linked to Rett syndrome, the leading cause of intellectual retardation in girls and causing mental, motor and growth impairment. Unstructured regions in MeCP2 provide the plasticity for establishing interactions with multiple binding partners. We present a biophysical characterization of the methyl binding domain (MBD) from MeCP2 reporting the contribution of flanking domains to its structural stability and dsDNA interaction. The flanking disordered intervening domain (ID) increased the structural stability of MBD, modified its dsDNA binding profile from an entropically-driven moderate-affinity binding to an overwhelmingly enthalpically-driven high-affinity binding. Additionally, ID provided an additional site for simultaneously and autonomously binding an independent dsDNA molecule, which is a key feature linked to the chromatin remodelling and looping activity of MeCP2, as well as its ability to interact with nucleosomes replacing histone H1. The dsDNA interaction is characterized by an unusually large heat capacity linked to a cluster of water molecules trapped within the binding interface. The dynamics of disordered regions together with extrinsic factors are key determinants of MeCP2 global structural properties and functional capabilities.

Among the thousands of proteins encoded in the human genome 30% of them are completely or partially devoid of stable structure^1^,^2^. These intrinsically disordered proteins (IDPs) are characterized by a global or local lack of secondary and tertiary structure, and they may undergo a structural rearrangement upon the interaction with their binding partners. This structural plasticity allows them to interact with a large variety of physiological partners (in fact, many IDPs are important hubs in protein interaction networks), adapting their conformation to different structural scaffolds. The presence of flexible regions facilitates structural rearrangements necessary for exposing different binding motifs and for allosteric regulation of binding partners. On the other hand, these interactions are characterized by a moderate-to-low binding affinity and a transient nature, because of the energetic penalty stemming from the conformational change required for the binding.

The structural effect of disordered regions in proteins is controversial. Intrinsically disordered regions have a priori unknown roles in molecular stability and function. While these regions are characterized by a biased residue composition, where polar and charged residues predominate and they exhibit a considerable propensity to be exposed to the solvent^3^, they still can make key contacts with structured regions and affect the global stability and the dynamics of the protein, as well as modulate the interaction with a binding partner. The impact of disordered regions on the global stability can be exerted through specific or unspecific effects. Specific effects may derive from long-lived or transient interactions between residues from disordered and structured regions, while unspecific effects may be due to reciprocal constrained flexibility/mobility of the polypeptide chain because of steric hindrance. Long-range electrostatic and dipolar interactions are extremely important in IDPs, especially at low ionic strength, because of the large fraction of charged and polar residues.

Prediction of IDP regions is usually made on the basis of the local structural and physico-chemical properties of the polypeptide chain^4^. Though these algorithms are quite robust, they may overestimate the extent of the disordered region^5^. On the other hand, there are many experimental techniques providing information about conformational changes coupled to binding interactions. Some of them provide structural information at an atomic (e.g. nuclear magnetic resonance) or molecular level (e.g. small-angle x-ray scattering), while some other provide detailed energetic information at a molecular level (e.g. isothermal titration calorimetry, ITC) compared to other less informative techniques. In particular, among other advantages, ITC allows the best estimation of the binding enthalpy, the binding stoichiometry, the heat capacity change upon binding, as well as the assessment of proton exchange events (or other additional equilibria) coupled to the binding interaction. Therefore, ITC provides the complete thermodynamic profile for any intermolecular interaction, from which valuable information can be extracted regarding the key structural and energetic determinants of such interaction.

Transcription regulation and chromatin architecture remodelling are two very complex processes tightly controlled by a huge number of proteins and epigenetic modifications, where slight alterations in the DNA or the proteins involved may result in disease. Rett syndrome (RTT) is an example of dysregulation of transcription and chromatin structure with severe consequences on neuronal development, differentiation and maturation. RTT is a disorder affecting 1/10000 live births. Although considered a rare disease, RTT is the main cause of mental retardation in females, characterized by a clinically varied expression and sharing features with other neurological autistic diseases. De novo mutations in the gene encoding Methyl-CpG binding Protein 2 (MeCP2) associated with an altered, defective MeCP2 protein (regarding its structural stability and folding, or its interaction with DNA and RNA or other protein partners) are involved in disease development^6^,^7^. MeCP2 is an IDP organized into six domains: N-terminal domain (NTD), methyl binding domain (MBD), intervening domain (ID), transcriptional repression domain (TRD), C-terminal domain α (CTDα), and C-terminal domain β (CTDβ) (Supplementary Fig. S1)^8^,^9^. In principle, the most important domains are MBD and TRD, initially associated with methylated CpG (mCpG) DNA binding or transcription repressor activities^10^,^11^ and the location for most of the mutations associated with RTT. However, it has been shown that other domains (ID, CTDα and CTDβ) are also directly or indirectly involved in methyl-independent DNA interaction^9^,^12^.

MBD is the best characterized domain in MeCP2 and its structure has been determined either free in solution or bound to mCpG-DNA^13^,^14^. Although there are some differences between these two structures, the wedge-shaped structured core can be described as a 3-stranded anti-parallel β-sheets with an α-helix on the C-terminal side. Flanking this core, two unstructured regions are found. Binding to DNA has been shown to increase MBD secondary structure. MBD is considered to be directly involved in maintaining full-MeCP2 organization through interactions with other domains. Therefore, it has been proposed to be the core of the protein structure as it would drive interdomain coupling, and mutations on this domain would impact on its own and global stability and function^8^.

Several mechanisms of action and interaction partners have been described for the MeCP2. MBD has been shown to provide strong selectivity through symmetrically methylated DNA duplex both *in vitro* and *in vivo*, modulated by environmental factors (in particular, ionic strength). In addition, MeCP2 has revealed broad distribution tracking the density of 5-methyl-cytidines, being especially abundant in the heterochromatin foci^15^. MeCP2 can also bind non-specifically to unmethylated DNA. Although this interaction shows lower affinity, that is not a large enough difference to explain its preferential distribution. Thus, it has been proposed that methylation density could modulate the specificity^16^. Similar to histone H1, MeCP2 is a chromatin-compacting factor in a methylation-independent manner^17^. Lack of MeCP2 results in a disorganized chromatin structure leading to impairment of synaptic plasticity derived from improper neuronal responses to stimuli. This represents a genome-wide, unspecific repressive activity, different from the specific repressor role associated with methylated DNA. Therefore, besides regulating the expression of specific genes, MeCP2 can act globally as a histone-like component organizing chromatin into a highly specialized organization^18^,^19^. Inverse correlation in MeCP2 and histone H1 levels, as well as their competitive binding to nucleosomes, indicates a compensatory mechanism between MeCP2 and histone H1 and points to a genome-wide function for MeCP2 with an architectural role similar to that of H1^18^. MeCP2 interacts with four potential nucleosomal binding targets: the free linker DNA through histone H1 displacement, the curved DNA wrapped around the nucleosome, the protein surface of the nucleosome, and the solvent exposed core histone N-terminal tail domains^16^,^20^. On the other hand MeCP2 interacts with other proteins involved in gene regulation, either co-repressors (e.g., mSin3A, cSki co-REST, NcoR/SMRT) for which the disruption of their interactions caused by RTT-associated mutations is a key element for understanding the pathology^21^,^22^, transcription factors (e.g., YY1) or activators (e.g., CREB)^15^,^23^, and it also interacts with RNA through an RNA-binding RG repeat region and regulates gene expression at a different level^24^.

These activities are related to different biological roles. MeCP2 is involved in gene regulation associated with either gene silencing or activation^25^. Several mechanisms are associated to this event, which is important for biological aspects such as decreasing transcriptional noise or adapting gene expression pattern to different physiological or environmentally-induced conditions^26^. In addition, MeCP2 acts as a chromatin architecture remodeller through interactions with nucleosomes, maintaining and reshaping local and global chromatin structures associated with gene expression regulation. Finally, MeCP2 participates in gene regulation at RNA level by interacting with RNA transcripts and RNA-binding proteins (e.g., YB1)^27^.

MeCP2 is a multifaceted protein where its structural and energetic properties must be intimately connected to its great variety of biological roles. The aim in this work is to reveal how some structural and double-stranded DNA (dsDNA) interaction properties provide the basis for some of these roles at a molecular level. In particular, the goal is twofold:

- To describe in detail the effect of flanking domains, NTD and ID, on the structural stability of MBD at low ionic strength. This has been done previously at high ionic strength^12^,^28^. However, it has been reported that the ability to discriminate between methylated and unmethylated dsDNA depends on ionic strength, among other factors^29^. Moreover, a highly polar, basic, disordered protein such as MeCP2 must be highly susceptible to its environment, especially at low ionic concentration where long-range electrostatic interactions may play a major role.
- To provide, for the first time, the complete thermodynamic profile of the interaction of MBD with dsDNA, assessing the role of the flanking domains (NTD and ID), as well as the effect of extrinsic factors such as the ionic concentration and the sequestering of water molecules within the polar protein-DNA interface^14^.

## Results

### Structural analysis of MeCP2 and variants

Far-UV CD spectra of MBD exhibited two regions typical from β-sheet and random-coil (centered around 208 nm) and α-helix (centered around 222 nm) (Supplementary Fig. S2), in agreement with the solution structure obtained by NMR (PDB code 1qk9) and the crystallographic structure obtained by x-ray diffraction (PDB code 3c2i)^13^,^14^. MBD is largely unstructured and, therefore, the intensity of the CD signal is small, as well as its change with temperature. Nonetheless, a small loss of secondary structure upon thermal denaturation could be observed. As expected, the far-UV spectra of the variants were similar in shape, but exhibited a lower intensity when normalized by the number of residues, indicating that the flanking domains are disordered. Differential scanning calorimetry experiments showed the same difficulties: the small structured core in MBD is associated to a low stability and a small molar unfolding enthalpy (low unfolding cooperativity), leading to a very small experimental signal. Increasing the protein concentration is impractical because MBD is prone to aggregation at high concentration.

**Figure 1:**
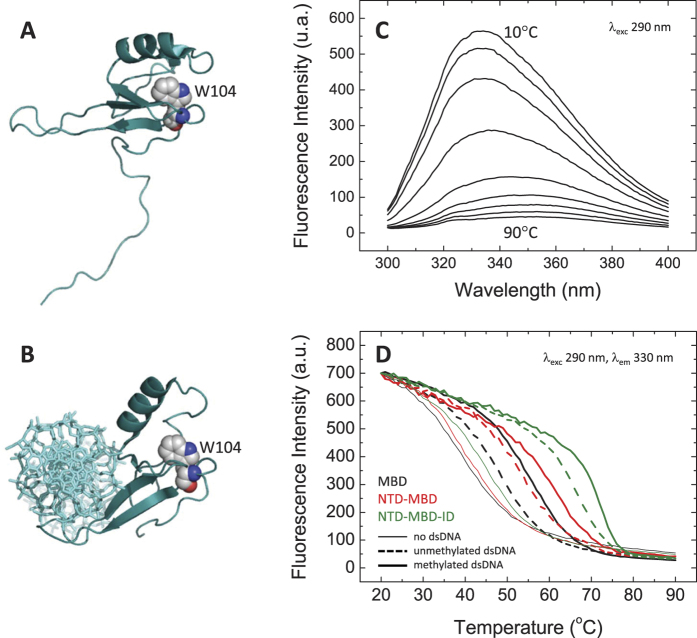
Structural features and thermal stability of the MeCP2 MBD. (**A**,**B**) Solution structure of MBD (pdb 1qk9) and crystallographic structure of MBD bound to mCpG-dsDNA (pdb 3c2i) showing the tryptophan 104 within the folding core. (**C**) Fluorescence spectra recorded at different temperatures showing the large quantum yield of the single tryptophan at low temperature and the substantial dynamic quenching by water molecules upon unfolding. (**D**) Fluorescence thermal denaturations for MBD, NTD-MBD and NTD-MBD-ID in the absence of dsDNA and in the presence of unmethylated and mCpG-dsDNA. All unfolding traces could be fitted considering a two-state unfolding model (not shown for clarity purposes). Figures have been created with PyMol (http://www.pymol.org/).

Fluorescence spectra monitoring the intrinsic fluorescence intensity of the single tryptophan residue in MBD (W104, Fig. 1) were employed to overcome the difficulties found in circular dichroism and differential scanning calorimetry. At low temperature MBD showed typical asymmetric bell-shaped spectra, indicating that the tryptophan residue is not solvent-exposed as observed in the NMR and crystallographic structures. A temperature increase caused a dramatic reduction in fluorescence intensity and a red-shift towards higher wavelengths (the wavelength for maximal intensity changed from 330 nm to 350 nm), due to dynamic water quenching of tryptophan fluorescence intensity when exposed to the solvent upon unfolding (Fig. 1). From a set of emission spectra in the temperature range 10–90 °C, the emission wavelength for the maximal spectral change was estimated to be 330 nm. The same behavior was observed with the variants.

### Thermal unfolding of MBD and variants

Thermal unfolding assays for MBD and its variants were performed by recording the fluorescence emission intensity at a fixed wavelength (330 nm) as a function of temperature (Fig. 1). Non-linear fitting assuming a two-state unfolding model allowed estimating the thermodynamic parameters that define the stability of MBD and its variants: midtransition temperature, *T*_*m*_, and unfolding enthalpy, Δ*H(T*_*m*_) (Table 1). Because thermal denaturations are rather insensitive to unfolding heat capacity values, Δ*C*_*P*_, an estimated value of 0.5 kcal/K·mol was employed according to published correlations between structural and energetic parameters^30^ and the known percentage of structure in MBD (60%)^12^; the excellent fits for the unfolding transitions validated that election. Reversibility tests confirmed the unfolding is fully reversible. In addition, the agreement with preliminary unfolding experiments using circular dichroism confirmed the applicability of the two-state unfolding model.

For MBD the *T*_*m*_ decreased with *pH* and increased with ionic strength, while the unfolding enthalpy (related to the folding/unfolding cooperativity level) did not change significantly. In general, if the stability of a protein increases (decreases) with the concentration of a co-solute, then, the folded (unfolded) state preferentially interacts with that co-solute. The following equation summarizes this phenomenon^31^,^32^:


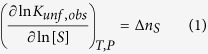


where *K*_*unf,obs*_ is the equilibrium constant for the unfolding process, *S* is the co-solute, and Δ*n*_*S*_ is the net number of co-solute molecules exchanged upon unfolding (positive for uptake or preferential interaction with the unfolded conformation, negative for release or preferential interaction with the native conformation). Assuming some approximations the following equation is obtained from equation (1)^32^,^33^:


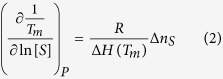


which can be employed to roughly estimate the number of co-solute molecules exchanged upon unfolding. Accordingly, the dependency of the stability with *pH* and ionic strength indicates that the unfolding process is coupled with the preferential interaction of protons and salt ions with either the folded or the unfolded conformations of MBD. Thus, because the stability of MBD decreases with increasing the *pH* (lowering concentration of protons), the folded MBD interacts preferentially with protons and its unfolding is coupled to the release of protons (that is, the unfolded MBD gets deprotonated, compared to the folded MBD). Similarly, because the stability of MBD increases with increasing the salt concentration, the folded MBD interacts preferentially with salt ions and the unfolding is coupled to the release of salt ions. In addition, salt ions may have an additional charge-screening effect and contribute to the increase stability at high ionic strength by diminishing repulsive interactions between positively charged groups. From the results in Table 1 and equation (1) it can be estimated that approximately −0.3 protons and −0.7 salt ions are released upon MBD unfolding. A rather similar behavior regarding the *pH* and ionic strength dependency was observed with the variants (Table 1).

The most striking result is that, at any *pH* and ionic strength, the structural stability (in terms of *T*_*m*_, and Δ*H(T*_*m*_)) gradually increased with the addition of the disordered domains NTD and ID. For example, at *pH* 7, the addition of NTD and ID increased the *T*_*m*_ in 2.3 °C and 7.8 °C, respectively. Therefore, those disordered regions contribute significantly, through specific or unspecific effects, to the structural stability of the molecule.

### dsDNA-induced stabilization effect on MeCP2 MBD and variants

The interaction of MBD and its variants with its physiological ligand was indirectly monitored by assessing the stabilizing effect induced by methylated and unmethylated dsDNA. As it has been indicated above, preferential interaction of a solute with the native conformation leads to stabilization of such conformation (equations (1) and (2)), which in practice can be observed as stabilization against thermal denaturation (increase in *T*_*m*_ and Δ*H(T*_*m*_)). Thermal denaturations were performed for MBD and its variants in the presence of unmethylated and mCpG dsDNA corresponding to the BDNF promoter region IV (Fig. 1), employing the same protocol used for the DNA-free proteins. The thermal denaturation curves were fitted using the two-state unfolding model and the apparent thermodynamic parameters for the unfolding of the protein-DNA complex were estimated.

In all cases dsDNA increased the stability of MBD, as observed in the values in *T*_*m*_ and Δ*H(T*_*m*_) compared to those for the dsDNA-free MBD, at each experimental condition (Fig. 1, Table 1). This is an indication of preferential binding of dsDNA with the folded MBD, as expected. The same results were observed for the protein variants.

The extent of the ligand stabilization effect (i.e. increase in *T*_*m*_, or Δ*T*_*m*_) on MBD depends on the binding affinity, the binding stoichiometry, and the concentration of dsDNA. Because the concentration of dsDNA was the same in all these assays, one would expect the Δ*T*_*m*_ values to be useful to rank binding affinities for different ligands (i.e., the larger the Δ*T*_*m*_ value, the higher the protein-dsDNA binding affinity). However, this is not the usual case, since the binding enthalpy and the binding heat capacity, which might be different for each ligand, also modulate the extent of the ligand-induced stabilization effect. In addition, domain ID can also interact with dsDNA^12^, leading to further stabilization of the native protein conformation.

Methylated dsDNA caused a stabilization effect on MBD larger than that of unmethylated DNA (Fig. 1, Table 1), reflecting the preferential interaction or specificity of MBD towards methylated DNA. The same phenomenon was observed for the variants including the flanking domains. Surprisingly, not only the flanking domains, NTD and ID, increase the thermal stability of dsDNA-free MBD, but they also enhance the stabilizing effect induced by the dsDNA binding. In fact, the stabilization effect of dsDNA on NTD-MBD-ID is much larger than that observed for the other proteins (Fig. 1, Table 1). The extent of the stabilization effect induced by dsDNA does not correlate with the measured binding affinities (see below), because there was very little difference in binding affinity between methylated and unmethylated dsDNA (see below). Very likely differences in the binding enthalpy and binding heat capacity might justify those distinctive different stabilization effects.

### Interaction of MeCP2 MBD and variants with dsDNA

Previous to the calorimetric study of the interaction of MBD with dsDNA, ultracentrifugation experiments were carried out in order to get information about the binding stoichiometry. Sedimentation velocity experiments provided sedimentation coefficients of 3.3S, 0.8S and 4.0S for the dsDNA, MBD and MBD-dsDNA complex, respectively, in agreement with their molecular mass in solution (Supplementary Fig. S3). Therefore, our results indicate a 1:1 protein:dsDNA binding stoichiometry for a 45 bp dsDNA fragment, as reported previously^10^, although a 2:1 protein:dsDNA stoichiometry has also been reported^26^.

The interaction of MBD and its variants with dsDNA was directly assessed by ITC. For the binding of MBD and NTD-MBD to dsDNA a model with a single binding site was considered, but for the binding of NTD-MBD-ID to dsDNA a model with two different binding sites had to be considered, since two distinguishable binding events could be clearly observed (see below).

**Figure 2:**
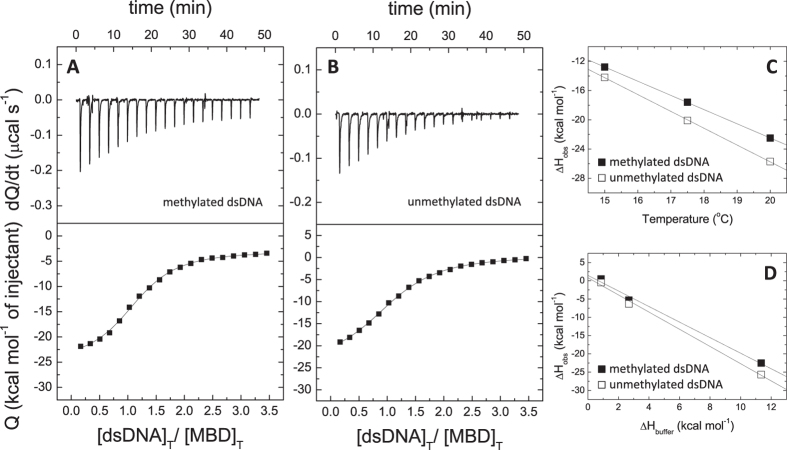
MBD interaction with dsDNA. (**A**,**B**) Calorimetric titrations of MBD interacting with dsDNA in Tris 50 mM, *pH* 7, 20 °C. The upper plots show the thermogram (thermal power as a function of time), whereas the lower plots show the binding isotherm (normalized heats as a function of the dsDNA/protein molar ratio). (**C**) Experiments at different temperatures provided an estimation of the binding heat capacity. (**D**) Experiments using buffers with different ionization enthalpy provided an estimation of the buffer-independent binding enthalpy and the net number of exchanged protons upon MBD-dsDNA complex formation.

The interaction between MBD and dsDNA was characterized by moderate affinity (dissociation constant in the submicromolar range), exhibiting an entropically driven binding with a binding enthalpy slightly unfavorable (Fig. 2, Table 2). Previous works reported higher affinities, but this can be reconciled considering the different experimental *pH* in our study. The interaction of MBD with dsDNA was coupled to the net release of 2 protons upon complex formation (therefore, at least two ionizable groups are involved in that proton exchange process) (Table 2). That means that increasing the *pH* in 0.5 units will increase the binding affinity 10-fold, resulting in a dissociation constant in fair agreement with published results^12^,^29^. In addition, the interaction of MBD with dsDNA is characterized by a large, negative binding heat capacity (Fig. 2, Table 2). MBD shows just a 2-fold difference in binding affinity in favor of methylated dsDNA. This small difference in dsDNA selectivity towards methylated DNA has been observed previously^29^. The increase in ionic strength to NaCl 150 mM significantly affected the binding affinity (∼1000-fold decrease) and could not be reliably determined (Supplementary Fig. S4). This binding affinity reduction indicates that, according to the following relationship^31^,^33^:


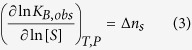


the formation of the complex is coupled to the release of approximately 4 salt ions from the complex (polyelectrolyte effect), in reasonable agreement with previously reported values^29^.

**Figure 3:**
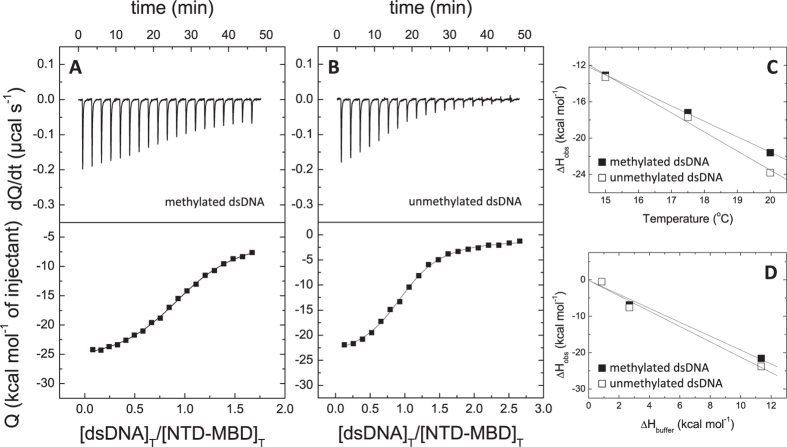
NTD-MBD interaction with dsDNA. (**A**,**B**) Calorimetric titrations of NTD-MBD interacting with dsDNA in Tris 50 mM, *pH* 7, 20 °C. The upper plots show the thermogram (thermal power as a function of time), whereas the lower plots show the binding isotherm (normalized heats as a function of the dsDNA/protein molar ratio). (**C**) Experiments at different temperatures provided an estimation of the binding heat capacity. (**D**) Experiments using buffers with different ionization enthalpy provided an estimation of the buffer-independent binding enthalpy and the net number of exchanged protons upon complex formation.

Addition of NTD to MBD did not change significantly the thermodynamic profile of the interaction with dsDNA (Fig. 3, Table 2). The only significant differences regarding MBD were: slightly higher binding affinity, a negligible binding enthalpy, and a slightly smaller binding heat capacity. Interestingly, the binding heat capacity for the binding of mCpG-dsDNA was always a bit smaller than that for the binding of unmethylated dsDNA.

**Figure 4:**
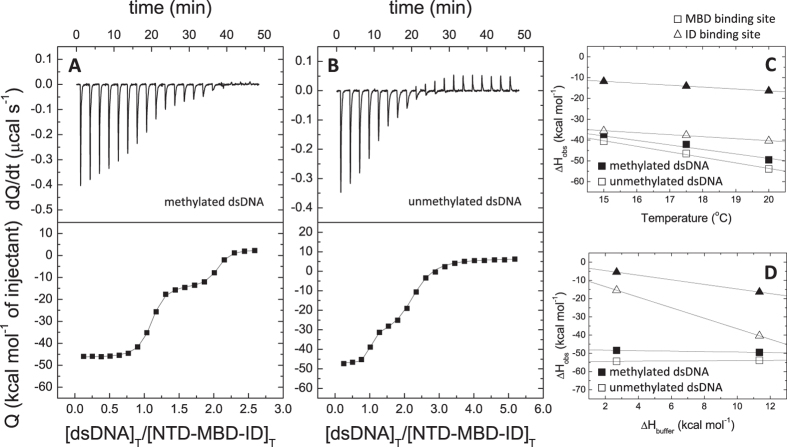
NTD-MBD-ID interaction with dsDNA. (**A**,**B**) Calorimetric titrations of NTD-MBD-ID interacting with dsDNA in Tris 50 mM, *pH* 7, 20 °C. The upper plots show the thermogram (thermal power as a function of time), whereas the lower plots show the binding isotherm (normalized heats as a function of the dsDNA/protein molar ratio). (**C**) Experiments at different temperatures provided an estimation of the binding heat capacities. (**D**) Experiments using buffers with different ionization enthalpy provided an estimation of the buffer-independent binding enthalpy and the net number of exchanged protons upon complex formation.

However the addition of ID changed completely the interaction, causing a dramatic increase in the binding affinity for dsDNA in MBD (dissociation constant changing from the submicromolar to the subnanomolar range), but it also provided an additional dsDNA binding site in ID (Fig. 4, Table 2). A preliminary analysis of the calorimetric titrations, using a model-free formalism considering two binding sites, indicated that both binding sites are different and independent^34^. This is a remarkable finding, provided that the ability of ID and MBD-ID to bind dsDNA was reported before^12^, but it was not established whether both binding sites would interact with the same dsDNA fragment or each domain would interact with an independent dsDNA fragment. Therefore, this work represents, to our knowledge, the first experimental evidence for two distinct, independent functional dsDNA binding sites in MBD-ID able to interact simultaneously with two independent dsDNA fragments. The presence of an additional dsDNA binding site is connected with the much larger dsDNA-induced stabilization effect observed on NTD-MBD-ID, compared to the stabilization effects on the other constructs (Fig. 1, Table 1).

The MBD binding site in the NTD-MBD-ID construct showed very high affinity (in the subnanomolar range) and a huge favorable binding enthalpy, with no net proton exchange (Table 2). This enthalpically driven binding was characterized by a large entropy loss that might reflect a large conformational reorganization upon dsDNA binding. On the other hand, the ID binding site showed lower affinity (in the submicromolar range) with favorable enthalpic and entropic contributions, and accompanied by a net proton release upon binding. Both binding sites exhibited a large, negative binding heat capacity. Interestingly, the binding heat capacity for the MBD binding site was much larger than that for the ID binding site, very likely reflecting a significant conformational rearrangement associated to the MBD binding (see Supplementary Fig. S2). In addition, the binding heat capacity for mCpG-dsDNA binding to MBD was a bit smaller than that for the binding of unmethylated dsDNA, but the binding heat capacity for mCpG-dsDNA binding to ID was similar to that of unmethylated dsDNA. Moreover, both MBD and ID sites display a slight higher selectivity (4-fold higher affinity) for mCpG-dsDNA.

## Discussion

### Structural stability of MBD

The *T*_*m*_ for MBD is rather low, as well as the unfolding enthalpy, indicating a moderate-to-low structural stability. In particular, an unfolding Gibbs energy (or stabilization energy) of 1.4 kcal/mol at 20 °C can be calculated from the values in Table 1, corresponding to a molar fraction of 8% unfolded protein. The reasoning underlying the selection of 20 °C as the reference temperature for the calorimetric titrations was straightforward: to keep a small percentage of unfolded protein, and to compare with a large body of published works on protein-DNA interactions studied by ITC. On the other hand, given the length of the dsDNA (45 bp), the stabilization energy of dsDNA is much larger than that of the protein. Thermal denaturation experiments performed with isolated dsDNA did not show unfolding of the dsDNA (hyperchromic effect) along the temperature range employed.

The estimation of the unfolding heat capacity as an adjustable parameter within the two-state unfolding model fitting analysis through spectroscopic denaturations is often not very reliable. However, it can be estimated as the slope in an enthalpy vs. temperature plot from a set of {*T*_*m*_, Δ*H(T*_*m*_)} pairs determined under slightly different conditions. Then, from the results in Table 1 an approximate value of 0.4 ± 0.3 kcal/K·mol is obtained for the unfolding heat capacity, which is reasonably close to the one employed in the data analysis.

The unfolding enthalpy provides an indication of the folding cooperativity and an indirect measurement of the amount of structured residues in the native conformation. Although with a large variability, it has been shown that the unfolding enthalpy at 60 °C, Δ*H*(60 °C), and 100 °C, Δ*H*(100 °C), of structured proteins correlate significantly well with the number of residues (*R*^2^ is 0.77 and 0.92, respectively)^30^. Thus, from the number of residues in MBD the expected Δ*H*(60 °C) and Δ*H*(100 °C) values are 60 and 110 kcal/mol, respectively; however, from the values in Table 1 the extrapolated Δ*H*(60 °C) and Δ*H*(100 °C) values are 40 and 60 kcal/mol. Therefore, the measured unfolding enthalpy is much smaller than the expected for a structured protein with the same molecular mass, and we may conclude that only 60% of MBD is structured, in complete agreement with previous experimental and computational results^12^,^28^.

### Interaction of MBD with dsDNA

From the experimentally determined thermodynamic profile for the MBD-dsDNA interaction (entropically driven binding, unfavorable binding enthalpy, large and negative binding heat capacity), it would be reasonable to expect that hydrophobic interactions are predominant. However, the binding interface between MBD and dsDNA is mostly of polar nature, with a majority of basic ionizable groups (Supplementary Fig. S5). It was noticed before that, in general, proteins binding to the major groove exhibit an enthalpically driven binding, whereas those proteins binding to the minor groove exhibit an entropically driven binding^35^. However, MBD binds to the major groove, but its binding is entropically driven, with a slightly unfavorable binding enthalpy.

**Figure 5:**
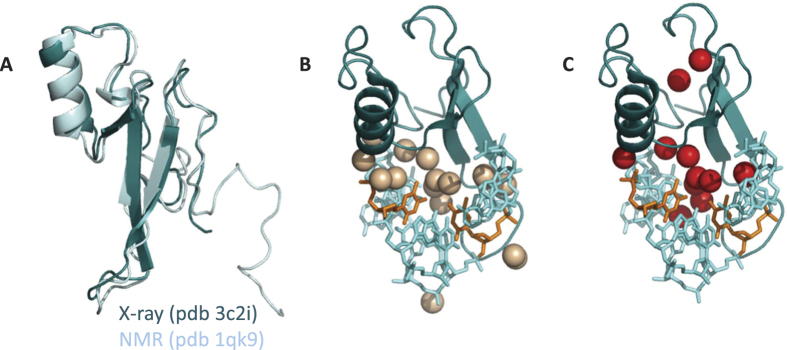
Conformational and hydration contributions to the MBD-dsDNA interaction. (**A**) Structural alignment of the structure of MBD in solution determined by NMR and the structure of MBD bound to mCpG-dsDNA determined by X-ray crystallography. (**B**) Network of hydrogen-bonding water molecules trapped in the MBD-dsDNA interaction interface. Water molecules at less than 4 Å simultaneously from both MBD and dsDNA are shown as spheres. Methyl-cytidines are shown in orange. (**C**) Buried hydrogen-bonding water molecules in the MBD-dsDNA complex. Water molecules with less than 2 Å^2^ of SASA are shown as spheres (SASA values were calculated with Surface Racer^70^). Most of the water molecules shown are completely buried (SASA = 0 Å^2^). Methyl-cytidines are shown in orange.

This thermodynamic binding profile for the MBD-dsDNA interaction is surprising for a number of reasons. The interaction interface between MBD and dsDNA is mostly polar (Supplementary Fig. S5) and specific polar interactions (hydrogen bonds, electrostatic interactions) are expected to be structurally and energetically predominant, which would result in a favorable binding enthalpy. Moreover, from the crystallographic structure of the MBD-dsDNA complex, the binding interaction results in the burial of 796 Å^2^ and 429 Å^2^ of polar and non-polar solvent-accessible surface area (SASA), that is, 65% and 35% of the total buried surface, respectively, and, therefore, desolvation upon binding mainly leads to the burial of polar surface area, which would result in a small, negative binding heat capacity. Moreover, being MBD largely disordered, a large conformational entropy penalty stemming from partial conformational rearrangement, which, together with a small desolvation entropy contribution derived from a very small non-polar surface desolvation, would lead to an unfavorable binding entropy. However, there are some observations supporting the unusual thermodynamic binding profile found for the MBD-dsDNA interaction. First, the alignment of the free-dsDNA and dsDNA-bound conformations of MBD indicates a very small conformational rearrangement (RMSD around 2 Å, either for all-atoms or α-carbon alignments) (Fig. 5). Second, disordered regions in MBD do not appear restructured in the dsDNA complex and do not refold upon interaction with dsDNA; in fact, several residues are missing in the crystallographic structure, with respect to the crystallized MBD, indicating those residues remain disordered and little refolding is occurring upon dsDNA binding. Therefore, the conformational entropic penalty should be rather small. Third, the burial of polar surface upon binding is not necessarily associated with a favorable binding enthalpy, because, if hydrogen bonds are not correctly established, the large enthalpic penalty coming from the desolvation of polar groups would contribute to an unfavorable binding enthalpy^36^. And fourth, there is a cluster of water molecules sequestered upon dsDNA binding and trapped in the MBD-dsDNA interface. These water molecules, involved in a hydrogen bond network between MBD and dsDNA, are highly restricted in a polar interface^14^ and they may contribute to a large, negative binding heat capacity (Fig. 5).

The interaction of MBD with dsDNA is characterized by a very small, unfavorable binding enthalpy. Thus, titrations performed using a buffer with a small ionization enthalpy (e.g. phosphate) are characterized by a small observed binding enthalpy, whereas titrations performed using a buffer with a large ionization enthalpy (e.g. Tris) are characterized by a large observed binding enthalpy. Then, the interaction of MBD with dsDNA is a nice example where the signal from the ligand binding becomes amplified through the beneficial coupling with the buffer protonation. It is important to emphasize that, provided that all precautions are taken, there are no bad buffers in isothermal titration calorimetry, and buffers with small and large ionization enthalpies are commonly employed for performing calorimetric titrations. Buffers with large ionization enthalpy may strongly contribute to the observed binding enthalpy, but that contribution can be conveniently removed for estimating the buffer-independent binding enthalpy and additional information can be obtained (e.g., Δ*n*_*H*_); moreover, the buffer contribution may compete with the intrinsic ligand binding enthalpy decreasing the observed signal or may amplify the observed signal. In addition (see Supplementary Information), the influence0 of the buffer on the determination of the ligand binding heat capacity is not negligible, but it is similar for Tris and phosphate (buffers with different ionization enthalpies and ionization heat capacities). Regarding the binding affinity, it is not affected by the buffer employed as long as the *pK*_*a*_ of the buffer is close to the experimental *pH*.

As it was observed previously, MBD exhibits low specificity regarding DNA methylation, since the binding affinity for mCpG-dsDNA is just 2-fold larger than that for unmethylated dsDNA and the binding enthalpy is very similar. However, significantly different stabilization effects are observed for methylated and unmethylated DNA (Δ*T*_*m*_ of 10 °C and 18 °C, respectively). These stabilization effects are much larger than those reported before^12^,^28^, but differences in the experimental *pH* and ionic strength may introduce differential effects. In addition, the stabilization effects reported here are not in agreement with the small binding affinity differences between mCpG- and unmethylated dsDNA, nor with the actual values of the dissociation constants. Because kinetic effects and slow assembly reorganization may play an important role (in a calorimetric titration we are observing the transient binding within the initial contact when a ligand approaches the receptor binding sites, but in a spectroscopic titration or in an unfolding assay we are observing the steady state binding after final accommodation of the ligand inside the receptor binding site), we are currently working on this issue.

The formation of the MBD-dsDNA complex is accompanied by the net release of about 2 protons. This strong *pH* dependency of the binding affinity (see equation (9), Methods) indicates that, in the vicinity of *pH* 7, a change in ±1 *pH* unit will increase/decrease the dissociation constant in a factor of 100. Those protons are dissociated from ionizable groups, belonging to MBD or dsDNA, undergoing a *pK*_*a*_ reduction upon dsDNA binding. Because MBD does not contain cysteines and histidines, and the *pK*_*a*_ of phosphates in the dsDNA is about 2, the only possible candidates are basic polar groups (tyrosines, arginines, and lysines) and acidic polar groups (aspartates and glutamates) (Supplementary Fig. S6). All these ionizable groups possess *pK*_*a*_ values quite far from the experimental *pH* and they would experience a *pK*_*a*_ reduction associated with the observed protons release only if their *pK*_*a*_ in the unbound state is abnormally low (for basic groups) or high (for acidic groups) and/or the *pK*_*a*_ change is very large. Thus, the identification of the ionizable groups directly involved in the proton exchange process is a matter of further work. However, importantly, the analysis of the calorimetric titrations performed using different buffers allowed the estimation of the buffer-independent binding parameters.

Calorimetric titrations performed at high ionic strength (NaCl 150 mM) showed a marked reduction in binding affinity (>1000-fold reduction) and could not be reliably determined (Supplementary Fig. S4), indicating that the formation of the MBD-dsDNA complex is coupled to the net release of salt ions. This diminished binding affinity is in agreement with the observed small dsDNA-induced stabilization effect in MBD at high ionic strength^12^. Additional calorimetric titrations performed under osmotic stress (glycerol 25%) showed a decrease in dsDNA binding affinity (4-fold reduction for mCpG-dsDNA and 2-fold reduction for unmethylated dsDNA) (Supplementary Fig. S4). Considering the following relationship^37^:


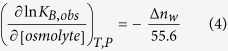


it can be estimated that the affinity loss caused by a reduction in water activity reflects the net uptake of water molecules (Δ*n*_*w*_ around 20 and 10 molecules for mCpG-dsDNA and unmethylated DNA, respectively) upon formation of the MBD-dsDNA complex. It is obvious that this must be a rather approximate number of water molecules (mostly associated with preferential hydration), since not all of them will undergo the same configurational constraint upon dsDNA binding.

### Influence of MBD-flanking domains

The behavior of the variants (NTD-MBD and NTD-MBD-ID) regarding their structural stability and the stabilizing effect of dsDNA binding is similar to that of MBD (Tables 1 and 2). In addition, the agreement with the previously published results is much better^12^,^28^, suggesting that the effect of the *pH* and ionic strength is much smaller for these variants.

It has been previously shown that, contrary to the results reported here, the addition of NTD and ID domains lowers the structural stability of MBD^12^. However, in those reported studies slightly different experimental conditions were employed (*pH* 7.4 and NaCl 150 mM). The different *pH* should not be responsible for the disagreement, since we observe a stabilization effect of the flanking domains in the *pH* range from 7 to 9. Therefore, the key factor must be the low ionic strength employed in our experiments. In a highly polar, basic protein, as MBD and its variants, the high ionic strength may screen specific and unspecific effects of the flanking disordered domains, resulting in a diminished stabilization effect.

The addition of the ID introduces a second dsDNA binding site, and, very important, it also dramatically enhances (400-fold) the affinity of the dsDNA-MBD interaction (Table 2, Fig. 4). As it has been mentioned above, the dsDNA binding capability of the ID is already known^12^, but it was not established whether MBD and ID would bind to the same dsDNA fragments or they would be able to bind two independent dsDNA fragments. Here two inflection points could be observed in the titrations with NTD-MBD-ID and it can be concluded that each domain can bind a different and independent dsDNA molecule (that is, MBD and ID do not interact with the same dsDNA molecule), which is an important finding supporting MeCP2 function as a chromatin architecture remodeling, dsDNA looping element, as well as its ability to interact with nucleosomes replacing histone H1^20^,^38^,^39^. The increase in binding affinity observed for MBD in the presence of ID indicates some kind of structural and energetic coupling between both domains.

The more plausible interpretation of the two binding sites observed in NTD-MBD-ID is that the high affinity site is located in MBD and the low affinity site is located in ID. This is consistent with: 1) submicromolar DNA affinity for isolated MBD and NTD-MBD; and 2) the DNA binding affinities reported for isolated MBD and isolated ID in a previous publication (higher affinity for MBD)^12^. On the other hand, preliminary data in our laboratory from MBD and NTD-MBD-ID variants carrying mutations located in MBD associated with RTT (R106W and R133C) show there is a dramatic change in the thermodynamic parameters associated with the binding site with higher affinity, while the binding site with lower affinity is hardly affected (data not shown). Because these mutations are located in MBD and the high affinity site is the only one affected, the high affinity site should correspond to MBD.

The isolated MBD interacted with dsDNA through an entropically-driven process with a small and unfavorable binding enthalpy; however, the MBD in the presence of ID showed a highly exothermic enthalpically-driven dsDNA binding with two large opposing enthalpic and entropic contributions (Table 2, Fig. 4), indicating that the mode of interaction for MBD with dsDNA is quite different depending on the MBD structural context. Substantial differences in the enthalpy-entropy partition of the Gibbs energy of binding have been linked to very different conformational changes, as well as allosteric effects, associated with the binding process^40^,^41^,^42^,^43^.

The two binding sites in NTD-MBD-ID showed significantly different binding affinities and enthalpies. While the ID binding site showed favorable enthalpic and entropic contributions, the MBD binding site showed an enthalpically driven binding with a considerable entropy loss, suggesting a large conformational change coupled to dsDNA binding. Regarding the binding heat capacity, the MBD binding site showed a very large negative value in all variants (with a more negative value for unmethylated dsDNA). Importantly, the MBD binding site in NTD-MBD-ID showed the largest binding heat capacity, very likely having a significant contribution from a conformational rearrangement coupled to dsDNA binding, while the ID binding site showed a smaller negative value (with a similar value for mCpG-dsDNA and unmethylated DNA) (Table 2). It has been reported that the isolated MBD undergoes a very small conformational rearrangement upon dsDNA interaction (from 60% to 66% in secondary structure), while the isolated ID shows a larger conformational rearrangement (from 38% to 59% in secondary structure)^12^. Therefore, the presence of the ID strongly affects, structurally and energetically, the dsDNA interaction at the MBD binding site.

In the NTD-MBD-ID the MBD binding site showed no net proton exchange upon dsDNA binding, whereas the ID biding site showed a net release of protons upon dsDNA binding. The interaction of dsDNA with NTD-MBD-ID also showed a polyelectrolyte effect. Similar to MBD interaction, the formation of the complex is coupled to the release of salt ions, and increasing the concentration of NaCl to 150 mM causes a ∼1000-fold reduction in the binding affinity. However, because the high affinity binding site has a dissociation constant in the subnanomolar range at low ionic strength (Table 2), the protein-dsDNA complex has a dissociation constant in the submicromolar range at high ionic strength and the binding affinity is still reliably determined by ITC (Supplementary Fig. S4).

### Water molecules involved in the MBD-dsDNA interaction

The large, negative binding heat capacity values associated with dsDNA binding deserve special attention, considering that, as already discussed, the binding interface is mostly polar and the dsDNA elicits very small conformational rearrangements. The observed binding heat capacity can be split into different contributions, each one stemming from any equilibrium coupled to dsDNA binding (see Supplementary Information). As explained in detail in the Supplementary Information, most of these contributions to the observed heat capacity are smaller than −0.2 kcal/K·mol and, therefore, they cannot explain the large, negative overall binding heat capacities (Table 2). There is an additional source for large negative heat capacities that is related to the early observation of a cluster of networking water molecules trapped within the MBD-dsDNA binding interface in the crystallographic structure^14^. Related to this observation, as indicated above, calorimetric titrations under osmotic stress suggest there is a net uptake of water molecules upon dsDNA binding. These water molecules located in an ordered environment establish hydrogen bonds between them, as well as between polar groups in the MBD and the dsDNA (Supplementary Fig. S7), and their highly restricted vibrational, librational and rotational modes lead to a reduction in their degrees of freedom associated which results in a large decrease in the heat capacity^44^,^45^,^46^,^47^,^48^. It has been estimated that a water molecule trapped within a polar protein-DNA interface may be associated to a reduction of up to −0.06 kcal/K·mol in the heat capacity. About 16 water molecules can be found at the binding interface between MBD and mCpG-dsDNA, at less than 4 Å from simultaneously both MBD and mCpG-dsDNA (Fig. 5). This value is very close to the value (20 water molecules) obtained from osmotic stress assays, and the difference could be due to additional loosely bound water molecules associated with the complex. Therefore, the local density of water molecules at the binding interface is 13 molecules/1000 Å^2^, larger than the average number for protein-protein interfaces (10 water molecules/1000 Å^2^) and similar to other protein-DNA interface^49^. It is intriguing that the interaction of MBD with unmethylated dsDNA is associated with a binding heat capacity slightly larger than that for mCpG-dsDNA, while the binding of unmethylated dsDNA seems to be coupled to the uptake of fewer water molecules.

## Conclusions

MeCP2 is a multifunctional protein involved in gene regulation and chromatin remodeling that specifically binds DNA and other protein partners. Most of the protein remains disordered under physiological conditions and that plasticity represents the structural and energetic basis for its multifunctional character. The structural and functional role of disordered regions is not obvious. However, the importance of those disordered regions becomes evident bearing in mind that some key mutations associated to RTT are located within those disordered regions. Clinically-relevant mutations in MeCP2 may alter its ability to fold and/or to interact properly with DNA or other proteins.

We have carried out the first comprehensive calorimetric study of MBD interacting with dsDNA. Additionally, we have performed a detailed characterization of different constructs including the N-terminal domain (NTD) and the intervening domain (ID) in order to shed light into the structural and functional role of these domains. We have assessed their contribution to the global stability and the interaction with dsDNA. From these results several important conclusions can be drawn:

- The inclusion of both disordered domains increases the structural stability and the folding cooperativity at low ionic strength.
- MBD and NTD-MBD possesses a single dsDNA binding site with moderate affinity (submicromolar dissociation constant), whereas NTD-MBD-ID possesses two dsDNA binding sites: a high affinity site (subnanomolar dissociation constant) and a moderate affinity binding site (submicromolar dissociation constant). To our knowledge, this is the first experimental evidence for two distinct and independent functional dsDNA binding sites in MBD-ID. Thus, NTD-MBD-ID is able to simultaneously attach two independent dsDNA fragments and this capability is part of the structural and energetic basis for MeCP2 involvement in chromatin architecture remodeling, looping activity and nucleosome interaction substituting histone H1.
- The thermodynamic profile for the interaction of MBD with dsDNA is remarkably unusual for a dsDNA major groove-binding protein. While MBD is significantly disordered and its interaction with DNA is mediated by polar residues, the interaction is entropically driven, characterized by a large negative binding heat capacity, and coupled to the release of protons and salt ions upon complex formation (heterotropic negative cooperativity).
- The inclusion of both domains, NTD and ID, increases the affinity of binding to CpG-methylated and unmethylated DNA. Because they also increase the stability of the dsDNA-free protein, the impact of both flanking domains on the stabilizing effect of CpG-methylated and unmethylated DNA is accumulative.
- The affinity enhancing effect of ID on the MBD binding site is considerably larger (>400-fold) compared to that exerted by NTD (3-fold).
- The presence of the ID alters dramatically the thermodynamic profile of the MBD binding site: while the isolated MBD shows an entropically-driven moderate binding affinity, the MBD within the NTD-MBD-ID construct shows an overwhelmingly enthalpically-driven high binding affinity.
- Both dsDNA binding sites in NTD-MBD-ID show markedly different thermodynamic profiles. In particular, the MBD site shows a high affinity interaction driven by a very large enthalpic contribution, whereas the ID site shows a moderate affinity interaction with favorable enthalpic and entropic contributions. Both binding sites exhibit a moderate selectivity for methylated dsDNA.
- The very large favorable binding enthalpy, the unfavorable binding entropy, and the very large binding heat capacity for the MBD binding site in the NTD-MBD-ID variant suggest a significant conformational rearrangement is coupled to the interaction with dsDNA.
- The observed large, negative binding heat capacity cannot be explained on the basis of the solvent-accessible surface area burial upon dsDNA binding. Moreover, the consideration of conformational changes (except for the NTD-MBD-ID variant, which very likely undergoes a significant conformational rearrangement upon dsDNA binding) and additional binding equilibria (protons and salt ions exchange) coupled to DNA binding do not justify the large binding heat capacity value. The network of hydrogen-bonded water molecules trapped between the protein and the dsDNA seems to be responsible for most of the large, negative binding heat capacity.

As a corollary from these conclusions, it can be established that the structural and functional properties of MBD from MeCP2 are dependent on the context. NTD and ID domains seem to play an important structural and functional role in MeCP2, and this adds to the rationale for its multifunctional nature, as well as the impact of mutations located within disordered regions. Therefore, intrinsic factors (presence of flanking domains, and dynamics and mobility of disordered regions), as well as extrinsic factors (ionic concentration and water molecules), strongly modulate the global structural properties and functional capabilities of MeCP2.

The key role of water molecules involved in the mCpG-dsDNA recognition by MBD has been postulated to be a general mechanism associated with mCpG recognition^50^, and, thus, the thermodynamic signature found for MeCP2 MBD should be a common general feature among mCpG recognition proteins. It is increasingly apparent that water is not just a passive matrix were physiological reactions take place, but water molecules are key active elements in many biomolecular processes such as protein folding^51^,^52^, nucleic acid assembly^53^, enzyme catalysis^54^, and molecular recognition^55^,^56^,^57^,^58^,^59^,^60^. In particular, waters at the interfaces of protein-DNA complexes may maintain packing density, screen electrostatic repulsions between charges, and act as linkers between complementary charges on the biomolecules^61^. Therefore, the thermodynamic profile of the binding energetics of a given interaction may be strongly affected by the active involvement of water molecules and it may contain a significant contribution from unusual hydration patterns.

There are some questions that must be addressed in future work: What are the structural roles of the other MeCP2 domains? How do they affect the structural and functional properties of MeCP2? What is the effect of mutations associated with RTT, located in structured or disordered regions, on the structural and functional properties of MeCP2? Why does unmethylated dsDNA induce a smaller stabilization effect on MBD while its thermodynamic binding profile is similar to that of mCpG-dsDNA? Given the larger binding heat capacity for unmethylated dsDNA binding, are water molecules also mediating that interaction? What are the thermodynamic binding parameters of isolated ID interacting with mCpG- and unmethylated dsDNA?

## Methods

### Plasmid construction

MBD and full-length human MeCP2 (isoform 2) were inserted in a pET30b plasmid for protein expression. The different protein variants were obtained by inserting appropriate stop codons: NTD-MBD and NTD-MBD-ID (Supplementary Fig. S1). The protein sequences contained an N-terminal polyhistidine-tag which was always removed after purification through an inserted PreScission Protease recognition cleavage site. All sequences were checked by sequencing analysis. The protein variants were checked and corroborated by Sanger sequencing using a BigDye Terminator v3.1 Cycle Sequencing Kit (Life Technologies, Carlsbad, CA) in an Applied Biosystems 3730/DNA Analyzer (Thermo Fisher Scientific, Waltham, MA). The data were analyzed with BioEdit Sequence Alignment Editor^62^.

### Protein expression and purification

All three protein variants (MBD, NTD-MBD and NTD-MBD-ID) were expressed and purified following the same procedure. Plasmids were transformed into BL21 (DE3) Star *E. coli* strain. Bacteria cultures were grown in 150 mL of LB/kanamycin (50 μg/mL) media at 37 °C overnight. Then, 4 L of LB/kanamycin (25 μg/mL) were inoculated (1:100 dilution) and incubated under the same conditions until reaching an OD (at a wavelength of 600 nm) of 0.6. Protein expression was induced with 1 mM isopropyl 1-thio-β-D-galactopyranoside (IPTG) at 18 °C overnight. Cells were ruptured by sonication in ice and benzonase (Merck-Millipore, Madrid, Spain) was added (20 U/mL) to remove nucleic acids. Proteins were purified using immobilized metal ion affinity chromatography (IMAC) in a HiTrap TALON column (GE-Healthcare Life Sciences, Barcelona, Spain) with two washing steps: buffer sodium phosphate 50 mM, *pH* 7, NaCl 300 mM, and in buffer sodium phosphate 50 mM, *pH* 7, NaCl 800 mM (to remove potential DNA contamination from the protein), before an imidazole 10–150 mM elution gradient. Purity was checked by SDS-PAGE.

Removal of the histidine-tag was performed by GST-tagged PreScission Protease processing in cleavage buffer (50 mM Tris-HCl, 150 mM NaCl, *pH* 7.5) at 4 °C for 4 hours. Progress of the protease processing was checked by SDS-PAGE. Finally, proteins were further purified using a combination of two affinity chromatographic steps to remove the histidine-tag (HiTrap TALON column, from GE-Healthcare Life Sciences, Barcelona, Spain) and the GST-tagged PreScission Protease (GST TALON column, from GE-Healthcare Life Sciences, Barcelona, Spain). Purity and homogeneity were checked by SDS-PAGE and size-exclusion chromatography. The proteins were stored in buffer Tris 50 mM *pH* 7.0 at −80 °C. The identity of all proteins was checked by mass spectrometry (4800plus MALDI-TOF/MS, from Applied Biosystems - Thermo Fisher Scientific, Waltham, MA). Potential DNA contamination was always checked determining the ration of UV absorption at 260 nm vs absorption at 280 nm. An extinction coefficient of 11460 M^−1^ cm^−1^ at 280 nm was employed for MBD and the variants.

Stability and binding assays were performed at different *pH* and buffer conditions (Tris 50 mM *pH* 7–9, NaCl 0–150 mM; Pipes 50 mM, *pH* 7; Phosphate 50 mM, *pH* 7). When needed, buffer exchange was performed using a 3 or 10 kDa-pore size ultrafiltration device (Amicon centrifugal filter, Merck-Millipore) at 4000 rpm and 4 °C.

### Double-stranded DNA

HPLC-purified methylated and unmethylated 45-bp single-stranded DNA (ssDNA) oligomers corresponding to the promoter IV of the mouse brain-derived neurotrophic factor (BDNF) gene^12^,^28^ were obtained from Integrated DNA Technologies. Two complementary pairs of DNA were used for DNA binding assays: forward unmethylated: 5′-GCCATGCCCTGGAACGGAACTCTCCTAATAAAAG-ATGTATCATTT-3′; reverse unmethylated: 5′-AAATGATACATCTTTTATTAGGAGAGTTCCGTTCC-AGGGCATGGC-3′; forward mCpG: 5′-GCCATGCCCTGGAA(5-Me)CGGAACTCTCCTAATAAA-AGATGTATCATTT-3′; reverse mCpG: 5′-AAATGATACATCTTTTATTAGGAGAGTTC(5-Me)CGTT-CCAGGGCATGGC-3′.

The DNA fragments were purchased as ssDNA oligonucleotides and they were subsequently annealed to obtain 45-bp double-stranded DNA (dsDNA) for the interaction experiments. Briefly, they were dissolved to obtain a 0.5 mM ssDNA solution for each oligonucleotide; then, they were mixed at an equimolar ratio and were annealed using a Stratagene Mx3005 P qPCR real-time thermal cycler (Agilent Technologies, Santa Clara, CA). The thermal annealing profile consisted of: 1) equilibration at 25 °C for 30 s; 2) heating ramp up to 99 °C; 3) equilibration at 99 °C for 60 s; and 4) 3-hour cooling process down to 25 °C at a rate of 1 °C/180 s.

### Circular dichroism

Circular dichroism spectra were recorded in a thermostated Chirascan spectrometer (Applied Photophysics, Leatherhead, UK) using a 0.1 cm path-length quartz cuvette (Hellma Analytics, Müllheim, Germany) with a bandwidth of 1 nm, an spectral resolution of 0.5 nm, and a response time of 5 s. Temperature was controlled by a Peltier unit and monitored using a temperature probe. The assays were performed in the far-UV range (190–260 nm). Protein concentration was set at 10–50 μM.

The poor signal related to the low content in secondary structure of the proteins and its small change during the thermal denaturation process within the temperature range 10–90 °C favored the use of fluorescence spectroscopy in the thermal unfolding assays.

### Fluorescence spectroscopy

Thermal unfolding studies were performed in a Cary Eclipse fluorescence spectrophotometer (Varian – Agilent, Santa Clara, CA) in three steps using a 1 cm path-length quartz cuvette (Hellma Analytics, Müllheim, Germany). Temperature was controlled by a Peltier unit and monitored using a temperature probe. Fluorescence emission spectra were recorded from 300 to 400 nm using an excitation wavelength of 290 nm and a bandwidth of 5 nm. Protein concentration was set at 5 μM.

Thermal stability assays were performed at a heating rate of 1 °C/min and at the wavelength for maximal spectral change. Thermal unfolding experiments were analyzed considering a two-state unfolding model:


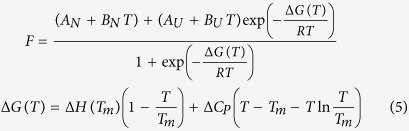


where *F* is the fluorescence signal, *T* is the absolute temperature, *T*_*m*_ is the midtransition temperature, Δ*H(T*_*m*_) is the unfolding enthalpy, Δ*C*_*P*_ is the unfolding heat capacity, and Δ*G* is the stabilization Gibbs energy. The adjustable parameters *A*_*N*_, *B*_*N*_, *A*_*U*_, and *B*_*U*_ define the pre- (native) and post-transition (unfolded) regions. This model can be applied to protein with low stability where the population of native protein is lower than 1 even at low temperature (that is, the straight line *A*_*N*_+*B*_*N*_*T* does not coincide with the observed signal), because the linear pre- and post-transition trends are fitted within the non-linear regression analysis procedure. The stabilizing effect upon dsDNA interaction was assessed performing thermal denaturations of the different proteins (at 5 μM) in the presence of methylated and unmethylated DNA (at 10 μM) under the same conditions.

### Isothermal titration calorimetry (ITC)

The interaction between the different proteins and dsDNA was characterized using an Auto-iTC200 microcalorimeter (MicroCal – Malvern Instruments, Malvern, UK). Protein in the calorimetric cell at 3–5 μM was titrated with dsDNA at 50 μM. All solutions were degassed at 15 °C for 2 min before each assay. A sequence of 2 μL-injections of titrant solution every 150 s was programmed and the stirring speed was set to 750 rpm. The association constant, *K*_*B,obs*_, and the enthalpy of binding, Δ*H*_*obs*_, were estimated through non-linear regression of the experimental data employing a single ligand binding site model (1:1 protein:dsDNA stoichiometry) or a two ligand binding sites model (1:2 protein:dsDNA stoichiometry) implemented in Origin (OriginLab, Northampton, MA)^34^,^63^.

Titrations were performed at different temperatures (15, 17.5 and 20 °C) in order to estimate the observed binding heat capacity change, Δ*C*_*P,obs*_:


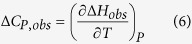


which is a parameter directly reflecting changes in solvent-exposed molecular surface upon protein-DNA complex formation and, therefore, it reflects conformational rearrangements coupled to binding. However, any equilibrium (e.g., ion release/uptake) coupled to ligand binding may contribute to the observed binding heat capacity. The narrow temperature range employed was appropriate, because the observed heat capacity change was remarkably large.

When ligand binding is coupled to proton exchange, the association binding constant, *K*_*B*_, is not affected by the buffer ionization as long as the *pK*_*a*_ of the buffer is close to the experimental *pH*, but it is influenced by the *pH* and the proton dissociation constants, *pK*_*a*_, of certain ionizable groups^31^,^64^:


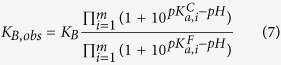


where *K*_*B*_ is the association constant for fully deprotonated reactants (at sufficiently high *pH*), *m* is the number of ionizable groups involved in the proton exchange (that is, those groups undergoing a *pK*_*a*_ change as a result of the complex formation), and *pK*_*a*_^*F*^ and *pK*_*a*_^*C*^ are the *pK*_*a*_ values for those ionizable groups in the free and the complex state. However, the observed binding enthalpy (and, therefore, the entropic contribution also) will contain an additional contribution from buffer ionization properties. In particular^64^,^65^,^66^:


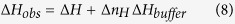


where Δ*H* is the buffer-independent enthalpy, Δ*H*_*buffer*_ is the ionization enthalpy of the buffer, and Δ*n*_*H*_ is the net number of exchanged protons between the protein-DNA complex and the bulk solution upon complex formation, which can be calculated as follows:


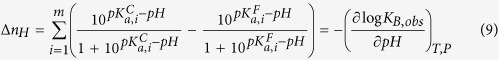


Thus, Δ*n*_*H*_ also indicates the change in binding affinity as a result of a change in *pH*. Titrations were performed in buffers with different ionization enthalpies (Tris, 11.35 kcal/mol; Pipes, 2.67 kcal/mol; and phosphate, 0.86 kcal/mol)^67^ in order to estimate the buffer-independent thermodynamic parameters (Δ*H* and Δ*n*_*H*_) from linear regression using equation (8).

### Analytical ultracentrifugation (AUC)

Sedimentation velocity assays were carried out at 48 krpm in an XL-I analytical ultracentrifuge (Beckman Coulter, Barcelona, Spain) equipped with UV-VIS absorbance and Raleigh interference detection systems. Sedimentation profiles were recorded at 260 nm. Sedimentation coefficient distributions were calculated by least-squares boundary modelling of sedimentation velocity data using the continuous distribution *c(s*) Lamm equation model as implemented by SEDFIT 14.1^68^. Experimental *s* values were corrected to standard conditions (water, 20 °C, and infinite dilution) using the program SEDNTERP to get the corresponding standard *s* values (*s*_*20,w*_)^69^.

Reported values always correspond to experimental replicates.

## Additional Information

**How to cite this article**: Claveria-Gimeno, R. *et al*. The intervening domain from MeCP2 enhances the DNA affinity of the methyl binding domain and provides an independent DNA interaction site. *Sci. Rep.* **7**, 41635; doi: 10.1038/srep41635 (2017).

**Publisher's note:** Springer Nature remains neutral with regard to jurisdictional claims in published maps and institutional affiliations.

## Supplementary Material

Supplementary Information

## Figures and Tables

**Table 1:** Unfolding stability parameters obtained from thermal denaturations followed by intrinsic tryptophan fluorescence^a^.

		*T*_*m*_(°C)	Δ*H(T*_*m*_) (kcal/mol)	Δ*T*_*m*_^*b*^(°C)
MBD	pH 7	38.4 ± 0.3	29 ± 1	
	pH 8	36.9 ± 0.3	33 ± 1	
	pH 9	30.8 ± 0.3	27 ± 1	
	pH 7, NaCl 150 mM	46.4 ± 0.4	32 ± 1	
	unmethylated dsDNA	48.9 ± 0.3	38 ± 2	10.5 ± 0.4
	methylated dsDNA	56.5 ± 0.3	44 ± 2	18.1 ± 0.4
NTD-MBD	pH 7	40.7 ± 0.2	33 ± 1	2.3 ± 0.4
	pH 8	39.3 ± 0.2	31 ± 1	2.4 ± 0.4
	pH 9	42.4 ± 0.2	34 ± 1	11.6 ± 0.4
	pH 7, NaCl 150 mM	48.0 ± 0.2	33 ± 1	1.6 ± 0.4
	unmethylated dsDNA	55.9 ± 0.2	42 ± 2	15.2 ± 0.3
	methylated dsDNA	62.6 ± 0.2	48 ± 2	21.9 ± 0.3
NTD-MBD-ID	pH 7	46.2 ± 0.2	37 ± 1	7.8 ± 0.4
	pH 8	45.9 ± 0.3	48 ± 3	9.0 ± 0.4
	pH 9	45.4 ± 0.2	53 ± 2	14.6 ± 0.4
	pH 7, NaCl 150 mM	49.8 ± 0.1	38 ± 1	3.4 ± 0.4
	unmethylated dsDNA	66.9 ± 0.1	61 ± 2	20.7 ± 0.2
	methylated dsDNA	71.2 ± 0.2	86 ± 4	25.0 ± 0.3

Experiments in the presence of mCpG- and unmethylated dsDNA were performed at *pH* 7.

^a^Unfolding stability parameters were estimated considering a two-state unfolding model. ^*b*^Differences in midtransition temperature, Δ*T*_*m*_, are calculated taking as a reference the *T*_*m*_ of the MBD at the same experimental conditions or the same variant in the absence of dsDNA.

**Table 2:** Buffer-independent dsDNA binding parameters obtained from calorimetric titrations at *pH* 7.

	dsDNA	*K*_*d*_^a^ (nM)	Δ*G*^b^ (kcal/mol)	Δ*H*^c^ (kcal/mol)	−*T*Δ*S*^d^ (kcal/mol)	Δ*C*_*P*_^e^ (kcal/K·mol)	Δ*n*_*H*_^*c*^
MBD	unmethylated	450	−8.5	0.8	−9.3	−2.3	−2.4
	methylated	240	−8.9	1.5	−10.4	−1.9	−2.1
NTD-MBD	unmethylated	210	−9.0	−0.2	−8.8	−2.1	−2.1
	methylated	90	−9.5	−0.2	−9.3	−1.7	−1.9
NTD-MBD-ID	unmethylated	1.9	−11.7	−54.6	42.9	−2.7	−0.1
		250	−8.9	−7.6	−1.3	−0.96	−2.9
	methylated	0.56	−12.4	−48.4	36.0	−2.2	−0.1
		62	−9.7	−2.1	−7.6	−0.92	−1.3

^a^*K*_*d*_ = (*K*_*B,obs*_)^−1^.

^b^Δ*G* = *RT* ln*K*_*d*_.

^c^Δ*H* and Δ*n*_*H*_ were estimated by performing titrations using buffers with different ionization enthalpies and through linear regression using equation (8).

^d^Entropic contribution was calculated according to: −*T*ΔS = Δ*G* − Δ*H*.

^e^Δ*C*_*P*_ was estimated by performing titrations at different temperatures and through linear regression using equation (6). Relative error in *K*_*d*_ is 10%; absolute errors in Δ*G* is 0.1 kcal/mol; absolute errors in Δ*H*, −*T*Δ*S* and Δ*C*_*P*_ are 0.3 kcal/mol; and absolute error in Δ*n*_*H*_ is 0.1.
