# Supplementary material for: The intervening domain from MeCP2 enhances the DNA affinity of the methyl binding domain and provides an independent DNA interaction site
Source: Sci Rep. 2017 Jan 31;7:41635. doi: 10.1038/srep41635 (PMC5282554; doi:10.1038/srep41635)
Supplement: Supplementary Information [file srep41635-s1.pdf]

SUPPLEMENTARY INFORMATION

The intervening domain from MeCP2 enhances DNA affinity of methyl binding domain and provides an independent DNA interaction site

Rafael Claveria-Gimeno, Pilar M. Lanuza, Ignacio Morales-Chueca, Olga C. Jorge, Sonia Vega, Olga Abian*, Manel Esteller*, Adrian Velazquez-Campoy*

Dissection of the different contributions to the dsDNA binding heat capacity

The large, negative binding heat capacity values associated with dsDNA binding deserve special attention, considering that, as already discussed, the binding interface is mostly polar and the dsDNA elicits very small conformational rearrangements. The observed binding heat capacity can be split into different contributions, each one stemming from any equilibrium coupled to dsDNA binding:

$$\Delta C_P = \Delta C_{P,B} + \Delta C_{P,0} + \Delta C_{P,C} + \Delta C_{P,S} + \Delta C_{P,other} \qquad (1)$$

where $\Delta C_{P,B}$ is the contribution to the binding heat capacity associated with the de/protonation of the buffer as a result of the proton exchange upon protein-dsDNA complex formation, $\Delta C_{P,0}$ is the contribution associated with the burial of solvent-accessible surface area with no conformational changes involved (direct apposition of the interacting molecules in the binding competent conformations), $\Delta C_{P,C}$ is the contribution associated with conformational changes and changes in internal mobility and vibrational degrees of freedom, $\Delta C_{P,S}$ is the contribution associated the exchange of solutes or co-ligands, e.g., protons and other ions (coupled equilibria or heterotropic interactions), and $\Delta C_{P,other}$ is the contribution associated with any other coupled event or equilibrium. The contribution to the binding heat capacity associated with the de/protonation of the buffer as a result of the proton exchange upon protein-dsDNA complex formation is given by (see equations (8) and (9), Materials and Methods):

$$\Delta C_{P,B} = \left(\frac{\partial \Delta n_H}{\partial T}\right)_P \Delta H_{buffer} + \Delta n_H \Delta C_{P,buffer} \qquad (2)$$

where ΔH_{buffer} is the ionization enthalpy of the buffer, $\Delta C_{P,buffer}$ is the ionization heat capacity for the buffer, and Δn_H is the net number of exchanged protons between the protein-dsDNA complex and the bulk solution upon dsDNA binding. In order to quantify that contribution we must estimate or delimit its value. The second term is easier to interpret and calculate: the larger the net number of protons exchanged and the larger the buffer ionization heat capacity, the larger the numerical value of this third term. The second term is a bit more complicated, since:

$$\left(\frac{\partial \Delta n_H}{\partial T}\right)_P = \sum_{i=1}^{m} \frac{10^{pK_{a,i}^C - pH}}{\left(1+10^{pK_{a,i}^C - pH}\right)^2} \frac{\Delta H_{H^+,i}^C}{RT^2} - \sum_{i=1}^{m} \frac{10^{pK_{a,i}^F - pH}}{\left(1+10^{pK_{a,i}^F - pH}\right)^2} \frac{\Delta H_{H^+,i}^F}{RT^2} \quad (3)$$

where $\Delta H^{C}_{H+,i}$, and $\Delta H^{F}_{H+,I}$ are the ionization enthalpies for the ionizable groups *i* involved in the proton exchange process in the complex and the free forms. On the other hand, applying the Maxwell's relations to the second heterotropic derivative of the binding equilibrium constant:

$$\frac{\partial}{\partial T}\left(\frac{\partial \ln K_{B,obs}}{\partial \ln[H^+]}\right)_P = \frac{\partial}{\partial \ln[H^+]}\left(\frac{\partial \ln K_{B,obs}}{\partial T}\right)_P \quad (4)$$

the following relationship is derived:

$$\left(\frac{\partial \Delta n_H}{\partial T}\right)_P = -\frac{1}{2.3RT^2}\left(\frac{\partial \Delta H_{obs}}{\partial pH}\right)_P \quad (5)$$

connecting the temperature derivative of the net number of protons exchanged upon binding with the *pH*-derivative of the observed binding enthalpy. Now, considering that the titrations for estimating the observed heat capacity were carried out in Tris buffer (ΔH_{buffer} and $\Delta C_{P,buffer}$ are 11.35 kcal/mol -0.014 kcal/K·mol, respectively) [1], the number of exchanged protons is around 2, the variation of the observed binding enthalpy with *pH* ($\Delta\Delta H_{obs}/\Delta pH$) would not be larger than e.g. 5 kcal/mol, and giving plausible values for the different parameters, the first and second terms in equation (2) are not larger than -0.14 kcal/K·mol and -0.028 kcal/K·mol, respectively. Therefore, the contribution of the buffer ionization to the observed binding heat capacity is not larger than -0.17 kcal/K·mol. Interestingly, if the titrations had been carried out using phosphate buffer, a buffer with much smaller ionization enthalpy (ΔH_{buffer} and $\Delta C_{P,buffer}$ are 0.86 kcal/mol -0.055 kcal/K·mol, respectively) [1], the contribution of the buffer ionization to the observed binding heat capacity is not larger than -0.13 kcal/K·mol, which is comparable to the contribution from Tris. Incidentally, it can be concluded that for estimating the binding heat capacity it is better to use a buffer with small ionization enthalpy and heat capacity; if not possible, a buffer with small ionization heat capacity is preferred if the number of exchanged protons is very large, but a buffer with small ionization enthalpy is preferred if the number of exchanged protons is very small.

The contribution $\Delta C_{P,0}$ associated with the burial of solvent-accessible surface area with no conformational changes involved (direct apposition of the interacting molecules in the binding

competent conformations) is usually parameterized as a function of polar and non-polar desolvation of solvent-accessible surface area (SASA) as follows:

$$\Delta C_{P,0} = a\Delta SASA_p + b\Delta SASA_{np} \quad (6)$$

where $\Delta SASA_p$ and $\Delta SASA_{np}$ are the changes in polar and non-polar SASA upon binding through direct docking of the interacting molecules in the binding competent conformations, and *a* and *b* are proportionality factors. Using standard parameterizations for protein stability and model compounds or protein-carbohydrate interactions [2,3], and considering the binding interaction results in the burial of 796 $Å^2$ and 429 $Å^2$ of polar and non-polar surface area, respectively, $\Delta C_{P,0}$ values of 0.014 kcal/K·mol or -0.22 kcal/K·mol are obtained.

The contribution $\Delta C_{P,C}$ associated with conformational changes and changes in internal mobility and vibrational degrees of freedom is given by [4-6]:

$$\Delta C_{P,C} = -\frac{K_{conf}}{1+K_{conf}}\Delta C_{P,conf} - \frac{K_{conf}}{(1+K_{conf})^2}\frac{\Delta H_{conf}^2}{RT^2} \quad (7)$$

where K_{conf} is the equilibrium constant for the conformational equilibrium between the unbound conformation and the binding-competent conformation, and ΔH_{conf} and $\Delta C_{P,conf}$ are the enthalpy and the heat capacity associated with the transition between those two conformational states, respectively. If the unbound conformation predominates ($K_{conf} >> 1$), then:

$$\Delta C_{P,C} \approx -\Delta C_{P,conf} \quad (8)$$

On the other hand, if the binding-competent conformation predominates ($K_{conf} << 1$):

$$\Delta C_{P,C} \approx 0 \quad (9)$$

If the unbound and the binding competent conformations are similarly populated ($K_{conf} \approx 1$), then:

$$\Delta C_{P,C} \approx -\frac{1}{2}\Delta C_{P,conf} - \frac{1}{4}\frac{\Delta H_{conf}^2}{RT^2} \quad (10)$$

For an intrinsically disordered protein becoming (partially) structured upon ligand binding the first situation would apply. Although MBD is considered to be 60% disordered, close examination and comparison of unbound and dsDNA-bound conformations reveal very little conformational differences between them and negligible refolding after dsDNA binding (Fig. 5 in main text). Therefore, although making a good estimation for $\Delta C_{P,C}$ is difficult, the value should not be large. In

fact, it has been previously determined that the MBD experiences a rather small ordering upon dsDNA binding (an increase in secondary structure from 60% to 66%) [7].

Finally, the contribution $\Delta C_{P,S}$ associated the exchange of solutes or co-ligands, e.g., protons and other ions (coupled equilibria or heterotropic interactions), is given by [4-6]:

$$\Delta C_{P,S} = -\sum_{i=1}^{m} \frac{K_{X,i}^{F}[X]}{1+K_{X,i}^{F}[X]} \Delta C_{P,X,i}^{F} + \sum_{i=1}^{m} \frac{K_{X,i}^{C}[X]}{1+K_{X,i}^{C}[X]} \Delta C_{P,X,i}^{C} - \sum_{i=1}^{m} \frac{K_{X,i}^{F}[X]}{\left(1+K_{X,i}^{F}[X]\right)^{2}} \frac{\left(\Delta H_{X,i}^{F}\right)^{2}}{RT^{2}} + \sum_{i=1}^{m} \frac{K_{X,i}^{C}[X]}{\left(1+K_{X,i}^{C}[X]\right)^{2}} \frac{\left(\Delta H_{X,i}^{C}\right)^{2}}{RT^{2}} \quad (11)$$

where *m* is the number of binding sites for the solute in MBD, $K^{F}_{X,i}$ and $K^{C}_{X,i}$ are the association constants for the binding of the solute (e.g., proton, ion, other ligand…, symbolized as *X*) to the dsDNA-free and dsDNA-bound MBD, $\Delta H^{F}_{X,i}$ and $\Delta H^{F}_{X,i}$ are the solute binding enthalpies for the dsDNA-free and dsDNA-bound MBD, and $\Delta C^{F}_{P,X,i}$ and $\Delta C^{C}_{P,X,i}$ are the solute binding heat capacities for the dsDNA-free and dsDNA-bound MBD. In particular, equation (11) for protons is written as follows:

$$\Delta C_{P,H^{+}} = -\sum_{i=1}^{m} \frac{10^{pK_{a,i}^{F}-pH}}{1+10^{pK_{a,i}^{F}-pH}} \Delta C_{P,H^{+},i}^{F} + \sum_{i=1}^{m} \frac{10^{pK_{a,i}^{C}-pH}}{1+10^{pK_{a,i}^{C}-pH}} \Delta C_{P,H^{+},i}^{C} - \sum_{i=1}^{m} \frac{10^{pK_{a,i}^{F}-pH}}{\left(1+10^{pK_{a,i}^{F}-pH}\right)^{2}} \frac{\left(\Delta H_{H^{+},i}^{F}\right)^{2}}{RT^{2}} + \sum_{i=1}^{m} \frac{10^{pK_{a,i}^{C}-pH}}{\left(1+10^{pK_{a,i}^{C}-pH}\right)^{2}} \frac{\left(\Delta H_{H^{+},i}^{C}\right)^{2}}{RT^{2}} \quad (12)$$

MBD interacting with dsDNA exhibits a net deprotonation (Δn_H around 2) coupled to dsDNA binding. Considering the possible groups undergoing deprotonation within the binding interface and plausible values for the proton interaction parameters ($pK^{F}_{a,i}$, $pK^{C}_{a,i}$, $\Delta C^{F}_{P,H+,i}$, $\Delta C^{C}_{P,H+,i}$, $\Delta H^{F}_{H+,i}$, and $\Delta H^{C}_{H+,i}$), the estimated value for the heat capacity associated to the exchange of each proton is not larger than -0.1 kcal/K·mol. In addition, MBD interaction with dsDNA is also coupled to the net release of salt ions, and the same line of reasoning applies.

Summarizing, there are several contributions to the observed heat capacity, and some of them (contributions from the buffer ionization, the burial of the solvent accessible surface area, and the exchange of protons and other ions) are not larger than -0.1 kcal/K·mol. The contribution from the conformational change can be considered small judging from the comparison between the unbound and dsDNA-bound conformations of MBD, but the constrained mobility and restricted vibrational

degrees of freedom associated to dsDNA binding are nor easily estimated. Hence, their contribution might be substantial, although not enough to explain the large negative observed binding heat capacities (Table 3 in main text).

There is an additional source for large negative heat capacities, within the remaining contribution $\Delta C_{P,other}$, that is related to the early observation of a cluster of networking water molecules trapped within the MBD-dsDNA binding interface [8].

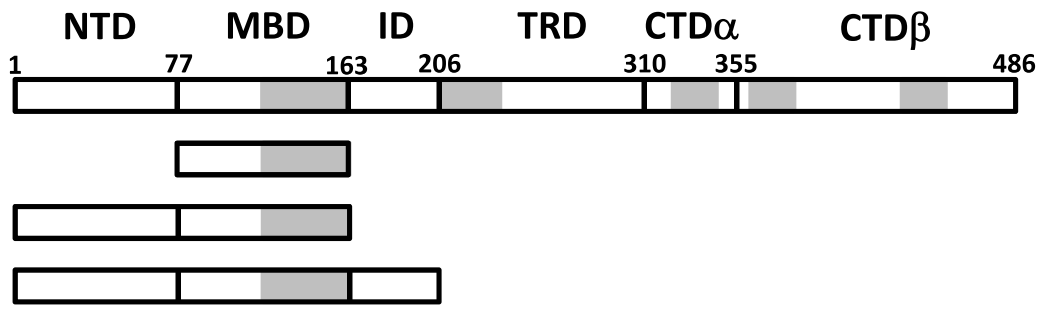

Figure S1. Domain structure in MeCP2. Schematic depiction of the domain organization in MeCP2. Structured regions are shown in grey. Numbers indicate the initial residue for each domain. Below the full-length MeCP2, the different constructs employed in this work are shown.

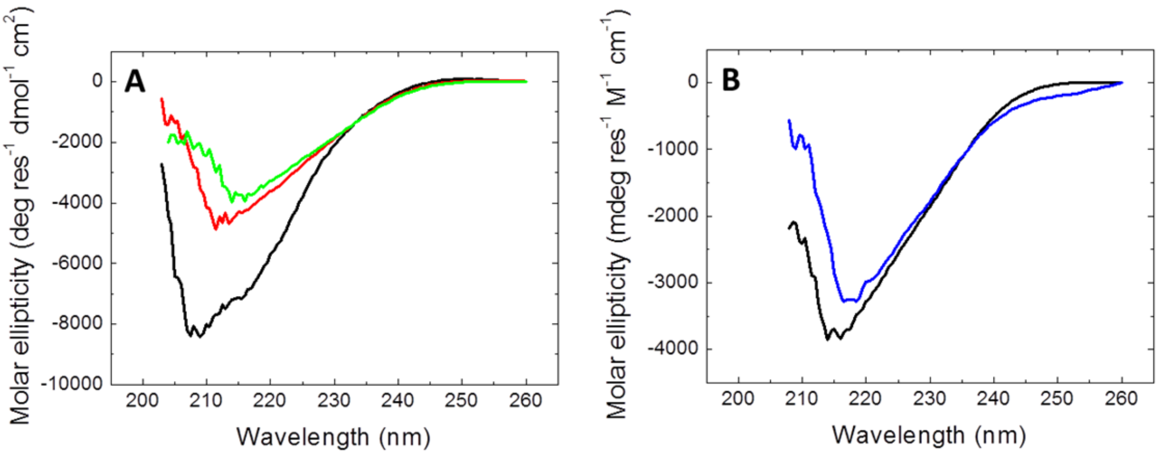

Figure S2. Structural features of MeCP2 MBD. (A) Circular dichroism spectra for MBD (black), NTD-MBD (red) and NTD-MBD-ID (green) in the absence of dsDNA. (B) Circular dichroism spectra for NTD-MBD-ID in the absence (black) and the presence of unmethylated dsDNA (blue). The contribution from dsDNA has been subtracted. Therefore, the dissimilarity between both spectra indicates a restructuring in the protein upon dsDNA binding.

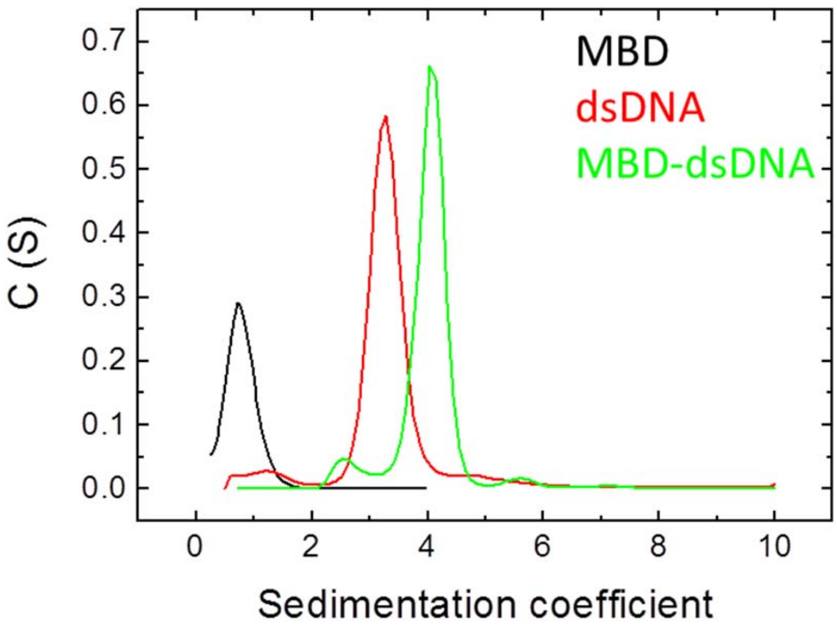

Figure S3. Apparent hydrodynamic behavior of MBD. Sedimentation coefficient distribution of MBD (black), unmethylated dsDNA (red), and MBD-dsDNA complex (green) in solution, in buffer Tris 50 mM, pH 7, 20°C.

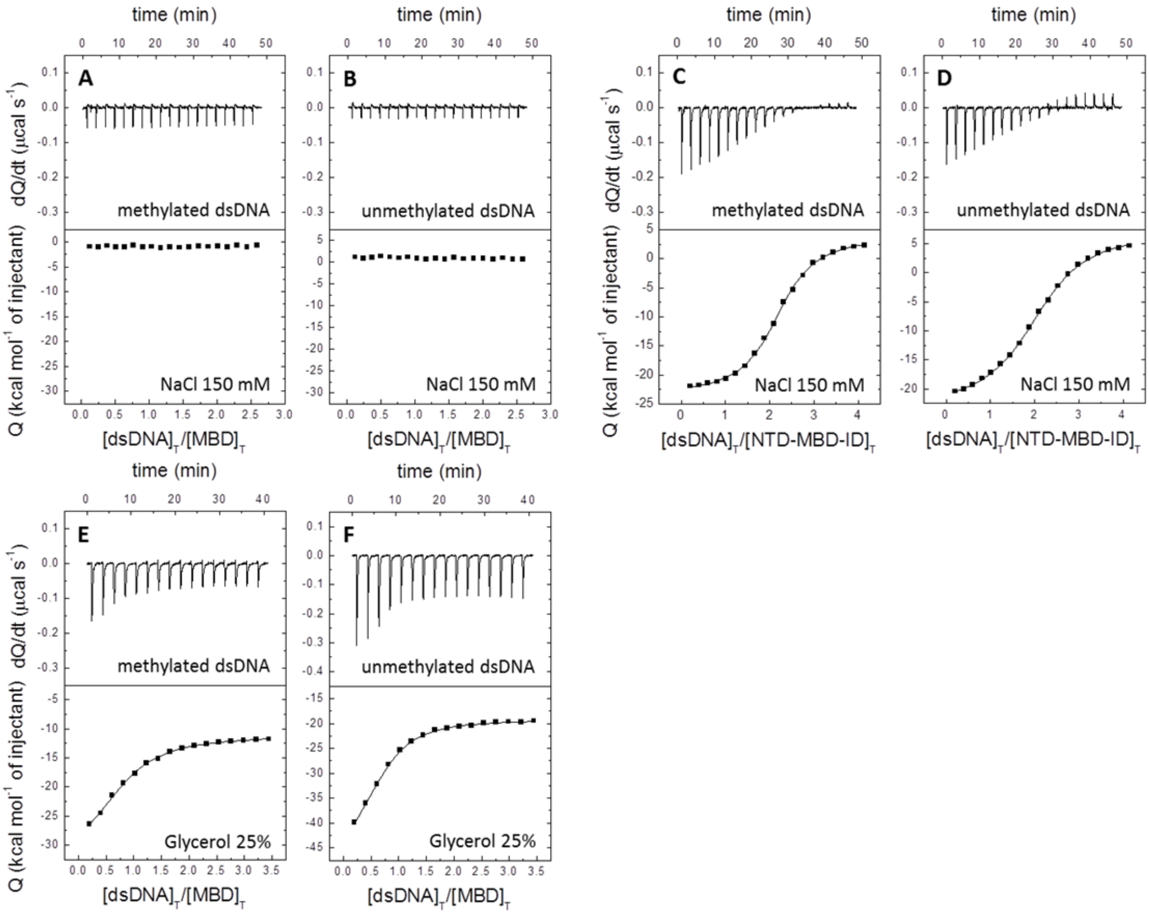

Figure S4. Ionic concentration and osmolyte effects on the MBD. Calorimetric titrations for dsDNA interaction in Tris 50 mM, *pH* 7, 20°C. The upper plots show the thermogram (thermal power as a function of time), whereas the lower plots show the binding isotherm (normalized heats as a function of the dsDNA/protein molar ratio. (A, B) Experiments performed with MBD in NaCl 150 mM. (C, D) Experiments performed with NTD-MBD-ID in NaCl 150 mM. (E, F) Experiments performed with MBD in glycerol 25%.

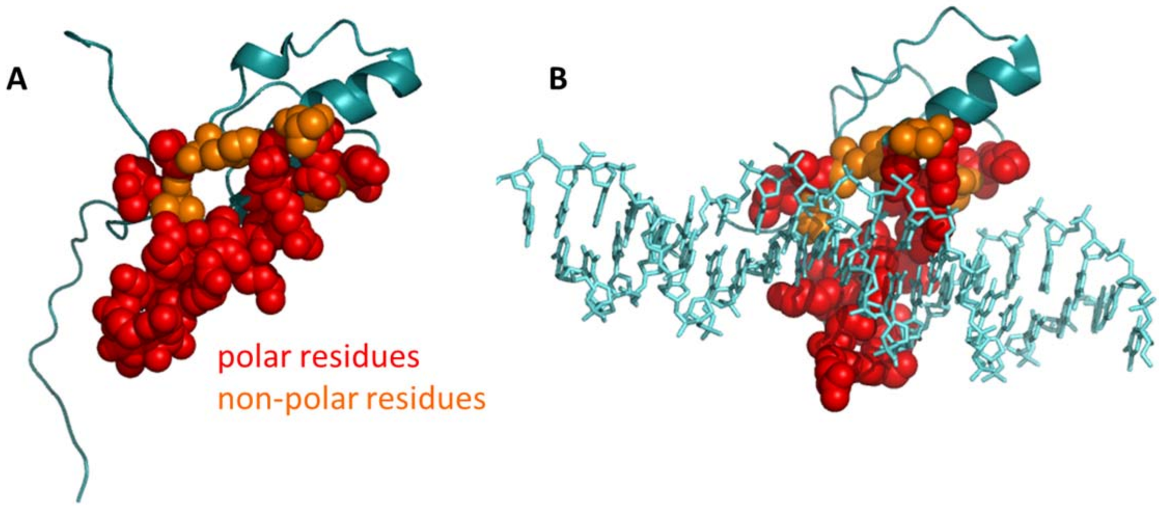

Figure S5. Polar character of the MBD binding interface. Comparison of the protein binding interface in the solution structure (A) and the crystallographic structure (B).

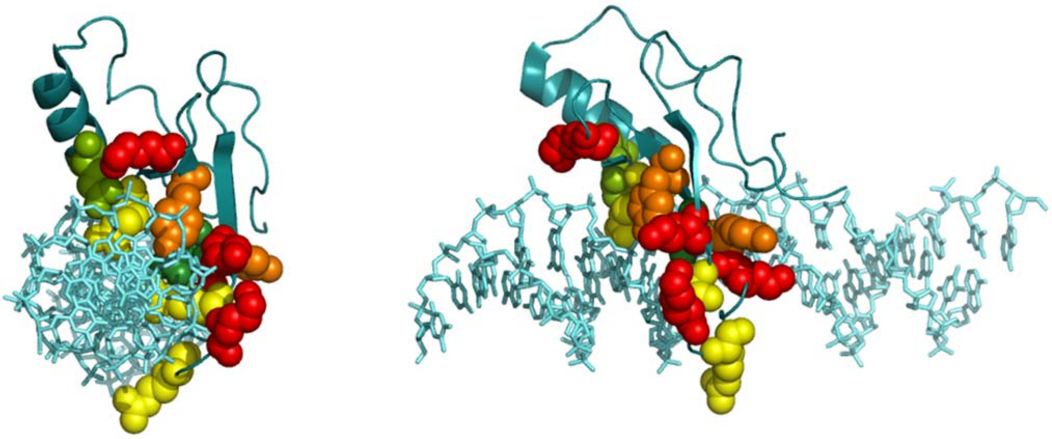

Figure S6. Ionizable groups at the MBD/mCpG-dsDNA binding interface. Two views of the dsDNA-bound structure of MBD showing the ionizable groups (at less than 4 Å from dsDNA) potentially involved in the net proton release upon complex formation: four lysines (red), three arginines (yellow), two tyrosines (orange), one glutamate (light green), and one aspartate (dark green) establish.

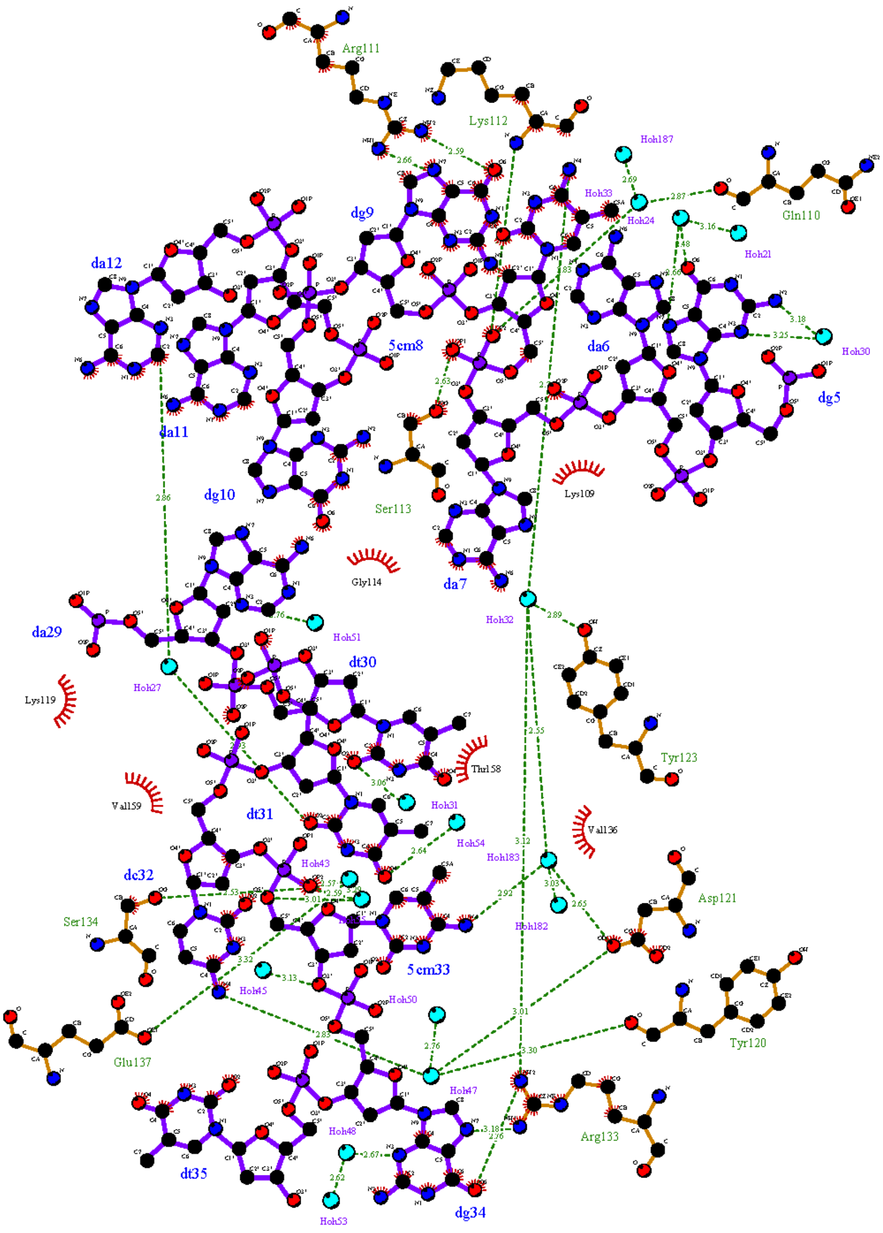

Figure S7. Interactions at the MBD/mCpG-dsDNA binding interface. Detailed depiction of the intermolecular interactions (hydrogen bonds and van der Waals interactions) between MBD and mCpG-dsDNA. Hydrogen-bonding water molecules are shown as blue spheres. Nucleotides 5-12 correspond to DNA chain B, whereas nucleotides 29-35 correspond to nucleotides 9-15 in chain C. This figure has been created with pdb 3c2i and LigPlot+ [9].

REFERENCES

1. Goldberg, R. N., Kishore, N. & Lennen, R. M. Thermodynamic quantities for the ionization reactions of buffers. *J. Phys. Chem. Ref. Data* **31**, 231-370 (2002).
2. Murphy, K. P. & Freire, E. Thermodynamics of structural stability and cooperative folding behavior in proteins. *Adv. Protein Chem.* **43**, 313-361 (1992).
3. Garcia-Hernandez, E. *et al*. Structural energetics of protein-carbohydrate interactions: insights derived from the study of lysozyme binding to its natural saccharide inhibitors. *Protein Sci.* **12**, 135-142 (2003).
4. Eftink, M & Biltonen, R. L. Thermodynamics of interacting biological systems, in *Biological Microcalorimetry* (ed. Beezer, A. E.) pp. 343-412 (Academic Press, 1980).
5. Eftink, M. R., Anusiem, A. C. & Biltonen, R. L. Enthalpy-entropy compensation and heat capacity changes for protein–ligand interactions: general thermodynamic models and data for the binding of nucleotides to Ribonuclease A. *Biochemistry* **22**, 3884-3896 (1983).
6. Vega, S., Abian, O. & Velazquez-Campoy, A. On the link between conformational changes, ligand binding and heat capacity. *Biochim. Biophys. Acta – Gen. Subjects* **1860**, 868-878 (2016).
7. Ghosh, R. P. *et al*. Unique physical properties and interactions of the domains of methylated DNA binding protein 2. *Biochemistry* **49**, 4395-4410 (2010).
8. Ho, K. L. *et al*. MeCP2 binding to DNA depends upon hydration at methyl-CpG. *Mol. Cell* **29**, 525-531 (2008).
9. Laskowski, R. A. & Swindells, M. B. LigPlot+: multiple ligand-protein interaction diagrams for drug discovery. *J. Chem. Inf. Model.* **51**, 2778-2786 (2011).
